# Polyfunctional incompletely differentiated CD8+ T cells accumulates after adoptive transfer of tumor-infiltrating lymphocytes and establish immunological memory in patients with metastatic melanoma

**DOI:** 10.1186/2051-1426-3-S2-P255

**Published:** 2015-11-04

**Authors:** Marco Donia, Julie Westerlin Kjeldsen, Rikke Andersen, Marie Christine Wulff Westergaard, Inge Marie Svane

**Affiliations:** 1 Center for Cancer Immune Therapy, Herlev Hospital, Copenhagen University, Herlev, Denmark

## Introduction

Infusion of highly heterogeneous populations of autologous tumor-infiltrating lymphocytes (TILs) can result in tumor regression of exceptional duration. Initial tumor regression has been associated to persistence of TILs shortly after infusion, but it is currently not clear whether a single infusion of TILs can establish long term memory antitumor responses.

## Methods

In order to characterize the long term fate of the infused tumor-reactive TILs and their attributes, we performed a longitudinal multidimension analysis of samples from 28 patients with metastatic melanoma treated in the trial NCT00937625 (TILs after lymphodepletion and followed by IL-2). The recognition of multiple autologous and allogeneic tumor antigens of TILs and peripheral blood lymphocytes (PBLs) collected serially up to 1 year after infusion was characterized at functional and phenotypic level by 13-colour FACS.

## Results

Frequency of T cell subpopulations and tumor recognition capacity of PBLs obtained shortly (1 week to 1 month) after infusion closely mirrored the characteristics of TILs infused in the individual patients. Bulk tumor-reactive T cells were detected in the circulation in the majority of patients with evidence of tumor regression (n=28, p < 0.01 vs patients with no tumor regression). Relative accumulation over time of tumor-reactive T cells simultaneously expressing TNF, IFN-g and CD107a indicated high persistence capacity of polyfunctional T cells. Combinatorial analysis of multiple surface markers (CD57, CD27, CD45RO, PD-1 and LAG-3) expressed on polyfunctional tumor-reactive T cells showed a unique pattern of T cell differentiation over time, with simultaneous accumulation of expression of the early differentiation marker CD27, alongside typical features of late effector cells such as loss of CD45RO and up-regulation of PD-1 and CD57 (combinatorial T cell differentiation score in n=15, PBMCs >1 month after infusion vs infusion products: p < 0.001).

## Conclusions

We demonstrated that TILs persisted in patients with tumor regression and identified a novel subset of tumor specific CD8+ T cells with an incomplete differentiation phenotype, co-expressing both early and late differentiation markers. This T cell subset has high capacity of persistence and generation of immunological memory in patients with metastatic melanoma.

